# Fish Meal Substitution Effects with the Combined Animal Proteins in the Feeds of Olive Flounder (*Paralichthys olivaceus*) on Growth Performance, Feed Availability, and Disease Resistance against *Streptococcus iniae*

**DOI:** 10.3390/ani14081162

**Published:** 2024-04-11

**Authors:** Yu Jin Shm, Sung Hwoan Cho, Taeho Kim

**Affiliations:** 1 Division of Convergence on Marine Science, Korea Maritime and Ocean University, Busan 49112, Republic of Korea; dbfl1543@naver.com; 2 Division of Marine Technology, Chonnam National University, Yeosu 59626, Republic of Korea; kimth@chonnam.ac.kr

**Keywords:** alternative protein source, blend of animal proteins, chicken by-product meal, *Paralichthys olivaceus*, challenge test

## Abstract

**Simple Summary:**

As fish meal (FM) has become an unsustainable natural resource in fish feeds because of its plateaued production and increased price, looking for a substitute for FM is a tremendous concern for scientists. Meat meal (MM) and chicken by-product meal (CBM) are known as suitable replacers for FM in the olive flounder (*P. olivaceus*) feeds. The combined proteins can substitute for more FM than each of the respective protein sources in diets because the former can compensate for their nutritional deficiency or imbalance. However, FM substitution with an alternative source in fish feeds may influence the immunity of fish. Therefore, this study evaluated the FM replacement effect of the combined MM and CBM (CMC) in the olive flounder feeds on growth, feed availability, and disease resistance against *S. iniae*. The results of this study indicated that FM up to 60% (39% FM protein in the diet) could be replaceable with CMC in the 65% FM-based diets of olive flounder without causing an unfavorable effect on growth performance, feed availability, or survival of fish after *S. iniae* infection.

**Abstract:**

This study aims to reveal the substitution impact of fish meal (FM) with the combined meat meal and chicken by-product meal (CMC) in the olive flounder (*P. olivaceus*) feeds on growth and feed availability. Seven experimental feeds were formulated. The control (CMC0) diet included 65% FM. In the CMC0 diet, the various (10%, 20%, 40%, 60%, 80%, and 100%) levels of FM were replaced with CMC, named as the CMC10, CMC20, CMC40, CMC60, CMC80, and CMC100 diets, respectively. The total number of 525 juvenile fish (9.2 ± 0.01 g; mean ± SD) was placed into 21 50-L flow-through tanks (25 juveniles/tank) with three replicates. Fish were hand-fed to apparent satiation for 8 weeks. After the 8-week feeding experiment, olive flounder fed the CMC10 (40.0 ± 0.60 g/fish, 2.99 ± 0.021%/day, and 39.57 ± 0.542 g/fish; mean ± SD), CMC20 (47.3 ± 2.58 g/fish, 3.24 ± 0.082%/day, and 45.16 ± 0.760 g/fish), and CMC40 (40.2 ± 1.17 g/fish, 3.00 ± 0.040%/day, and 39.43 ± 0.930 g/fish) diets attained superior (*p* < 0.0001 for all) weight gain, specific growth rate (SGR), and feed consumption compared to olive flounder fed the CMC0 (35.1 ± 0.96 g/fish, 2.81 ± 0.039%/day, and 33.75 ± 0.544 g/fish), CMC60 (31.7 ± 1.62 g/fish, 2.66 ± 0.068%/day, and 31.60 ± 1.080 g/fish), CMC80 (24.7 ± 0.63 g/fish, 2.33 ± 0.033%/day, and 25.27 ± 0.689 g/fish), and CMC100 (17.8 ± 0.32 g/fish, 1.92 ± 0.021%/day, and 18.99 ± 0.592 g/fish, respectively) diets. Weight gain, SGR, and feed consumption of olive flounder fed the CMC60 diet were comparable to olive flounder fed the CMC0 diet. Feed efficiency and protein efficiency ratio of olive flounder fed the CMC60 diet (1.02 ± 0.007 and 1.79 ± 0.034) were comparable to fish fed the CMC0 diet (1.04 ± 0.012 and 1.85 ± 0.021, respectively). None of the plasma and serum measurements, proximate composition, amino acid profiles, or survival of olive flounder after *S. iniae* infection were influenced by dietary treatments. In conclusion, CMC can substitute FM up to 60% (39% FM protein in the diet) without deteriorating growth performance, feed availability, or the survival of fish after *S. iniae* infection.

## 1. Introduction

Olive flounder (*P. olivaceus*) is an endemic fish species distributed from the Western Pacific Ocean to Japan’s Kuril Islands and the South China Sea and is a popular flat fish in Eastern Asia, particularly in the Republic of Korea [1]. It is a predatory fish that feeds primarily on mysid crustaceans during its larval and juvenile stages and preys on vertebrates such as wild fish as it grows [2]. The total aquaculture production of olive flounder was ca. 45,839 metric tons (MT), accounting for about 51% of the total marine aquaculture production (91,105 MT) in Korea in 2022 [3] and covering over 96% of the world’s production in 2022 [4]. Accordingly, aquaculture of olive flounder is playing an important role in Korea.

Aquaculture techniques will advance steadily with the increasing world population [5] and the growing demand for seafood for human consumption. In considering the upsurge in the global aquaculture industry, the growth of aquaculture will be largely driven by feeds for efficiency in nutrient transfer [6]. About 68% of fish and crustaceans depend on fish meal (FM)-based commercial feed, and FM has been used as the most popular protein source in feeds for aquatic animals [7]. FM contains complex nutrients, such as essential amino acids (EAA), essential fatty acids (EFA), trace elements, minerals, and good palatability and high digestibility [6,7]. In particular, commercial feeds for olive flounder contain more than 60% FM [8,9] because they require over 50% dietary protein [10,11]. However, FM is an unpromising protein resource for aquaculture because it is no longer sustainable and its price is rocketing on a periodic basis [12]. Therefore, fish farmers are recognizing that FM reduction in fish feeds is very essential for sustainable aquaculture [13].

Over the last decades, animal by-products have been extensively utilized as substitutes for FM in the feeds of several fish, including olive flounder [14], hybrid grouper (*Epinephelus fuscoguttatus* × *E. lanceolatus*) [15], and rockfish (*Sebastes schlegeli*) [16], because not only do they usually contain high protein and good EAA, but they are also available year-round and less expensive than FM [6,17]. Since about 50% of animal by-products are generated during the slaughter process [18] and more than 100 million MT are produced annually [19], they can be regarded as important replacers for FM in fish feeds in the future.

Among animal by-product meals, meat meal (MM) is derived from cattle, swine, and poultry and manufactured by rendering inedible or non-marketable by-products from the slaughter process [20]. MM had superior or comparable digestibility to FM in feeds for carnivorous species, such as rainbow trout (*Oncorhynchus mykiss*) [21] and rockfish [22], indicating MM has high potential as a FM replacer in fish feeds. MM can replace FM up to 80% (44% FM protein in the diet) in the 55% FM-based feed without compromising growth performance and proximate composition of the whole-body rockfish when rockfish were fed with a 55% FM-based diet or one of the diets replacing 10, 20, 40, 60, 80, and 100% FM with MM (68.2% crude protein) without AA supplementation [16]. In particular, analyzing proximate composition of the whole-body fish is a way to assess whether the fish attains the right nutrients to estimate survival and normal growth. Furthermore, 20% (13% FM protein in the diet) and 40% (26% FM protein in the diet) FM with low (65% crude protein) [14] and high (80% crude protein) quality of MM [23] were successfully substituted in the 65% FM-basal feeds of olive flounder without deteriorating growth or feed availability. In addition, FM up to 60% (48% FM protein in the diet) can be replaced by MM in the 80% FM-based diet without causing an unfavorable effect on the growth of olive flounder [24].

Poultry by-product meal (PBM) is manufactured by rendering the head, feet, neck, and internal organs, excluding feathers, commonly generated in poultry processing plants [20]. It has been used as the pet feed as well as fish feed [25] because of its high crude protein content, abundance of EAA, minerals, vitamins, and high palatability [26]. Fifty percent FM (29% FM protein in the diet) could be replaceable by PBM supplemented with the limiting AA (lysine and methionine) without causing any negative impact on growth performance when juvenile gilthead sea bream (*Sparus aurata*) were fed with a 58% FM-based diet or one of the diets replacing 25% FM by PBM with or without the limiting AA supplementation or 50% FM by PBM with AA supplementation [25]. The poultry slaughtering species in Korea are chicken and duck, and the slaughtering rate of chicken accounts for about 94% of the total PBM in Korea in 2022 [27]. Chicken by-product meal (CBM), which is made by drying and grinding chicken carcasses, including fat-trimmed lean meat, can be utilized as a replacement for FM because of its high crude protein and lipid content and cheap price [28,29]. In particular, Nandakumar et al. [29] unveiled that FM up to 28.6% (10% FM protein in the diet) could be substitutable with chicken waste meal in a 35% FM-basal diet, deteriorating the growth and feed efficiency (FE) of seabass (*Lates calcarifer*). Lately, Ha et al. [28] also proved that CBM can substitute 50% FM (32.5% FM protein in the diet) in diets without detrimental effects on growth, feed availability, plasma and serum parameters, or the chemical composition of the whole-body olive flounder when fish were fed with a 65% FM-basal diet or one of the diets substituting various (10–50%) levels of FM with CBM for 56 days.

Despite the potential of individual animal protein sources, the single protein as a FM substitute is still limited due to its low EAA content compared to FM. MM has a low EAA content, except for arginine [21,23], and CBM has a low content of histidine, isoleucine, lysine, methionine, tryptophan, and valine among EAA in comparison to FM [28]. The combined use of diverse animal by-product meals can generally replace a higher amount of FM than a respective protein source in diets because of the improved nutritional values of the respective alternative source by compensating for nutritional deficiency or imbalance [30,31]. Gunathilaka et al. [32] suggested that the combined MM and CBM (CMC) was an effective substitute for FM in the feeds of red sea bream (*Pagrus major*) when fish were fed with diets substituting half the amount of FM with corn gluten meal, soy protein concentrate, MM, CBM, and their combinations for 8 weeks. Ye et al. [15] also unveiled that 80% FM (56% FM protein in the diet) could be replaceable with the mixture of PBM, spray-dried blood meal (SBM), and shrimp meal in the juvenile hybrid grouper diets containing 70% FM. In addition, the combined PBM, meat and bone meal (MBM), blood meal, and feather meal could replace half the amount of FM in malabar grouper (*Epinephelus malabricus*) feeds without producing an unfavorable impact on growth performance [33].

Dietary FM substitution with an alternative protein source might affect the immunity of fish [34,35]. Therefore, nutritional trials of fish were often concluded with a bacterial challenge test, serving as a final indicator of the health status of fish [35,36]. *Streptococcus iniae*, a Gram-positive bacterium, is a pathogen that causes streptococcosis in fish and is one of the most critical aquatic pathogens leading to the high mortality of olive flounder in aquaculture in Korea [37,38] and worldwide [39].

A single source of MM [23] or CBM [28] is known as the effective replacement of FM in the olive flounder diet, but their combination effect has not been known yet. This study, therefore, aims to reveal the effect of diverse levels of FM substitution with CMC in the feeds of olive flounder on growth, feed availability, biochemical composition, plasma and serum measurements, and a challenge test against *S*. *iniae*.

## 2. Materials and Methods

### 2.1. Preparation of the Experimental Feeds

The CMC is the combined MM (Daekyung Oil & Transportation Co., Ltd., Busan, Republic of Korea) and CBM (Charmfre Co., Ltd., Buan-gun, Jeollabuk-do, Republic of Korea) at a ratio of 1:1, and each ingredient was purchased from local distributors. Seven isonitrogenous (56.5%) and isolipidic (10.8%) feeds were created (Table 1) to meet the dietary requirements of olive flounder [10,11]. The control diet (CMC0) included 65% FM and 12% dehulled soybean meal as the protein sources, 2.3% each of fish and soybean oils as the lipid sources, and 14.9% wheat flour as the carbohydrate source, respectively. The various (10%, 20%, 40%, 60%, 80%, and 100%) levels of FM in the CMC0 diet were substituted by CMC, named as the CMC10, CMC20, CMC40, CMC60, CMC80, and CMC100 diets, respectively.

All ingredients with water at a ratio of 3:1 were thoroughly blended, and the pellets were made using a lab pellet extruder (Dongsung Mechanics, Busan, Republic of Korea) (diameters of die in 4 and 6 mm). The experimental feeds were dried using an electronic drying machine (SI-2400; SIN IL Drying Machine Co., Ltd., Daegu, Republic of Korea) at 40 °C for 24 h and then kept in a freezer at −20 °C until use.

### 2.2. Rearing Conditions

Juvenile olive flounders of similar sizes were purchased from a private hatchery (Taean-gun, Chungcheongnam-do, Republic of Korea). Before beginning the feeding trial, olive flounder were adapted to the trial conditions by being fed with a commercial pellet (Suhyup Feed, Uiryeong-gun, Gyeongsangnam-do, Republic of Korea) containing 54% crude protein and 8% crude lipid for 10 days. After the 10-day adaptation period, 525 juvenile olive flounder (initial weight of 9.2 ± 0.01 g; mean ± SD) were randomly placed into 21, 50-L flow-through tanks (25 fish/tank) with 3 replicates. Olive flounder were carefully hand-fed to apparent satiation twice a day (08:30 and 17:30) for 8 weeks. The satiety of fish was decided at the point at which fish voluntarily stopped eating. The amount of diet supplied to each tank was measured daily. The bottoms of tanks were siphon-cleaned daily, and dead fish were immediately removed on observation. The photoperiod was not controlled but followed natural conditions, and all tanks were continuously provided with sand-filtered seawater at a flow rate of 3.5 L/min/tank. The water quality was measured using multiparameter water quality meters (AZ-8603; AZ Instrument Corp., Taichung, Taiwan) daily. The water temperature changed from 16.2 to 24.0 °C (20.1 °C ± 2.04 °C; mean ± SD), dissolved oxygen changed from 7.3–10.4 mg/L (9.3 ± 0.74 mg/L), salinity changed from 31.9 to 33.1 g/L (32.5 ± 0.70 g/L), and pH changed from 7.9 to 8.2 (8.0 ± 0.08), respectively.

### 2.3. Measurements of the Biological Indices of Fish

After the 8-week feeding period, all live fish from each tank were fasted for 24 h. Afterwards, fish were anesthetized using tricaine methanesulfonate (MS-222) at 100 ppm and counted and weighed from each tank collectively. Five anesthetized olive flounder from each tank were randomly chosen to assess their biological indices. The assessment of growth performance, feed availability, and biological indices of fish was conducted using the following formulas: specific growth rate (SGR, %/day) = [Ln final weight of fish (g) − Ln initial weight of fish (g)] × 100/days of the feeding trial (56 days), FE = weight gain of fish/feed consumption, protein efficiency ratio (PER) = weight gain of fish/protein consumption, protein retention (PR, %) = protein gain of fish × 100/protein consumption, condition factor (K, g/cm^3^) = body weight of fish (g) × 100/total length of fish (cm^3^), viscerosomatic index (VSI, %) = viscera weight of fish × 100/body weight of fish, and hepatosomatic index (HSI, %) = liver weight of fish × 100/body weight of fish.

### 2.4. Plasma Analysis of Fish

Plasma samples were collected using a heparinized syringe from the caudal vein of 3 randomly chosen anesthetized fish from each tank after the 8-week feeding trial. Then, the plasma samples were extracted by centrifuging the blood samples at 2,700× *g* for 10 min at 4 °C and kept in a freezer at −70 °C for measurements of aspartate aminotransferase (AST; original reference code 15809542), alanine aminotransferase (ALT; 16654035), alkaline phosphatase (ALP; 16653964), total bilirubin (TBL; 16654061), total cholesterol (TCO; 16654073), triglyceride (TRG; 16654085), total protein (TPT; 16654097), and albumin (ALB; 16653952) using an automatic chemistry system (FUJI DRI-CHEM NX500i; FUJIFILM Corp., Tokyo, Japan).

### 2.5. Serum Analysis of Fish

Serum samples were collected using a syringe from the caudal fin of 3 randomly chosen anesthetized olive flounder from each tank. Afterwards, the serum samples were extracted by centrifuging blood samples at 2,700× *g* for 10 min at 4 °C and stored at −70 °C until use. Lysozyme activity was measured as the amount of enzyme required to lyse *Micrococcus lysodeikticus*, based on Lange et al.’s [40] study. In brief, 100 µL of serum was blended with a suspension of 1.9 mL of *M. lysodeikticus* (0.2 mg/mL; Sigma-Aldrich Inc., St. Louis, MO, USA) in 0.05 M sodium phosphate buffer (pH 6.2). The reaction was incubated at 25 °C, and the absorbance was read at 530 nm at 15 min intervals for 60 min.

SOD was evaluated using a 19160 SOD determination kit (Sigma-Aldrich Inc., St. Louis, MO, USA) by measuring the reduction of a water-soluble tetrazolium salt (WST-1) to form a water-soluble formazan dye in the presence of a superoxide anion. The methods and procedures for measuring the SOD of the samples were the same as in Li et al.’s [41] study.

### 2.6. Biochemical Composition of the Samples

All experimental feeds, 10 initial fish before the feeding trial, and all surviving (≥3) olive flounder from each tank after the completion of the 8-week feeding experiment were homogenized to evaluate biochemical composition. Moisture, crude protein, crude lipid, and ash contents of the samples (the experimental diets and whole-body fish) for proximate composition were analyzed according to the standard method [42]. The moisture content of the experimental feeds and the whole body of olive flounder were dried in a dry oven at 105 °C for 4 and 24 h, respectively. The crude protein and crude lipid content of the samples were determined using the Kjeldahl (Kjeltec^TM^ 2100 Distillation Unit; Foss, Hillerød, Denmark) and ether extraction (Soxtec^TM^ 2043 Fat Extraction System; Foss, Hillerød, Denmark) methods, respectively. The ash content of the samples was determined using a muffle furnace at 550 °C for 4 h.

AA of the samples, except for tryptophan, were measured using an AA analyzer (L-8800 Auto-analyzer; Hitachi, Tokyo, Japan), and tryptophan content was measured using HPLC (S1125 HPLC pump system; Sykam GmbH, Eresing, Germany). The fatty acids (FA) of the samples were confirmed by comparison with the samples of known standards (37-component FAME mix CRM47885; Supelco™, St. Louis, MO, USA). Lipids for FA analysis of the samples were obtained with a blend of chloroform and methanol (2:1 *v*/*v*) based on Folch et al.’s [43] study. The methods and procedures for AA and FA analysis of the samples were the same as Jeong et al.’s [44] study.

### 2.7. Challenge Test of Fish

After the completion of the 8-week feeding period, 10 olive flounder in each tank were re-fed with the same designated feeds as the 8-week feeding experiment for 7 days to minimize the stressors. After that, 10 fish from each tank were used for the challenge test against *Streptococcus iniae* (FP5024). Olive flounder were artificially injected through intraperitoneal injection with 0.1 mL bacteria at 8.2 × 10^6^ CFU/mL, according to Ha et al.’s [23] study. The mortality of olive flounder was observed for the following 8 days after the *S. iniae* infection; mortality was monitored at a 6 h interval for the first 4 days and a 12 h interval for the last 4 days. Dead fish were removed from each tank on observation. Each tank was kept static for 8 days, and fish were unfed during the 8-day post-observation period.

### 2.8. Statistical Analysis

Data were subjected to one-way ANOVA and Tukey’s honestly significant difference test after normality (Shapiro–Wilk’s test) and homogeneity of variances (Levene’s test) using SPSS version 24.0 (SPSS Inc., Chicago, IL, USA). All values were presented as the mean ± standard deviation (SD) of the mean. Before statistical analysis, percentage data were arcsine-transformed. Statistical differences were regarded as significant with a *p*-value < 0.05. For statistically significant data, regression analysis was conducted to fit a suitable model. *p*-value was used for optimal regression selection to estimate an appropriate CMC inclusion level for the dependent variable. Survival of olive flounder was analyzed during the 8-day post-observation period after *S. iniae* infection by using the Kaplan–Meier survival curve, Log-Rank, and Wilcoxon tests.

## 3. Results

### 3.1. AA and FA Profiles of the Experimental Feeds

All EAA contents in FM, except for arginine, were relatively high over those in CMC (Table 2). Arginine content increased with dietary increased FM replacements with CMC but decreased for all other EAAs. Arginine, lysine, and threonine content in all experimental diets appeared to meet the dietary requirements of olive flounder [45,46,47], but methionine contents in all experimental feeds appeared to be lower than the dietary requirements [48].

The total contents of saturated FA (∑SFA) and monounsaturated FA (∑MUFA) in CMC were relatively high over those in FM, but low for the total content of n−3 highly unsaturated FA (∑n−3 HUFA) (Table 3). The contents of eicosapentaenoic acid (EPA; C20:5n−3) and docosahexaenoic acid (DHA; C22:6n−3) were not detected in CMC. The content of ∑SFA and ∑MUFA increased with dietary increased FM replacement with CMC, but decreased for ∑n−3 HUFA. The content of ∑n−3 HUFA in the CMC80 and CMC100 diets did not meet the dietary ∑n−3 HUFA requirement of olive flounder [49].

### 3.2. Survival, Weight Gain and SGR of Olive Flounder

Survival of olive flounder varied from 97.3 to 100%, but it was not remarkably (*p* > 0.1) changed by dietary treatments (Table 4). The greatest weight gain (47.3 ± 2.58 g/fish; mean ± SD) and SGR (3.24 ± 0.082%/day) were attained in olive flounder fed the CMC20 diet. Furthermore, weight gain and SGR of olive flounder fed the CMC10 (40.0 ± 0.60 g/fish and 2.99 ± 0.021%/day) and CMC40 (40.2 ± 1.17 g/fish and 3.00 ± 0.040%/day) diets were statistically (*p* < 0.05) greater than those of fish fed the CMC0 (35.1 ± 0.96 g/fish and 2.81 ± 0.039%/day), CMC60 (31.7 ± 1.62 g/fish and 2.66 ± 0.068%/day), CMC80 (24.7 ± 0.63 g/fish and 2.33 ± 0.033%/day), and CMC100 (17.8 ± 0.32 g/fish and 1.92 ± 0.021%/day) diets. Weight gain and SGR of olive flounder fed the CMC60 diet were comparable to those of olive flounder fed the CMC0 diet but statistically (*p* < 0.05) greater than those of fish fed the CMC80 and CMC100 diets. In regression analysis, cubic relationships were shown to be the most fit model between dietary increased FM replacement with CMC (X) vs. weight gain (Y = 0.000117X^3^ − 0.021899X^2^ + 0.852379X + 35.0989, R^2^ = 0.9560, *p* < 0.0001, Y_max_ = X value of 24.1%) and SGR (Y = 0.000003662X^3^ − 0.000756X^2^ + 0.030304X + 2.8084, R^2^ = 0.9711, *p* < 0.0001, Y_max_ = X value of 24.4%), respectively (Table 5).

### 3.3. Feed Availability and Biological Indices of Fish

Feed consumption (45.16 ± 0.760 g/fish; mean ± SD) of olive flounder fed the CMC20 diet was significantly (*p* < 0.0001) higher than that of olive flounder fed all other diets (Table 6). Furthermore, feed consumption of olive flounder fed the CMC10 (39.57 ± 0.542 g/fish) and CMC40 (39.43 ± 0.930 g/fish) diets was significantly (*p* < 0.05) higher than that of fish fed the CMC0 (33.75 ± 0.544 g/fish), CMC60 (31.60 ± 1.080 g/fish), CMC80 (25.27 ± 0.689 g/fish), and CMC100 (18.99 ± 0.592 g/fish) diets. Feed consumption of olive flounder fed the CMC60 diet was comparable to fish fed the CMC0 diet but statistically (*p* < 0.05) higher than that of fish fed the CMC80 and CMC100 diets. The cubic relationship in regression analysis was shown to be the most fit model between dietary increased FM replacement with CMC (X) vs. feed consumption (Y = 0.000102X^3^ − 0.019279X^2^ + 0.748248X + 35.0246, R^2^ = 0.9608, *p* < 0.0001, Y_max_ = X value of 24.0%). 

FE (1.05 ± 0.040) of olive flounder fed the CMC20 diet was statistically (*p* < 0.03) higher than that (0.99 ± 0.020) of fish fed the CMC100 diet, but not statistically (*p* > 0.05) different from that of fish fed the other diets. The PER of olive flounder fed the CMC0 (1.85 ± 0.021), CMC10 (1.81 ± 0.011), CMC20 (1.85 ± 0.071), and CMC40 (1.81 ± 0.022) diets was statistically (*p* < 0.002) higher than that of fish fed the CMC100 diet (1.69 ± 0.074), but not statistically (*p* > 0.05) different from that of fish fed the CMC60 (1.79 ± 0.034) and CMC80 (1.74 ± 0.029) diets. Cubic relationships were shown to be the most fit model between dietary increased FM replacement with CMC (X) and FE (Y = −0.0000002692X^3^ − 0.00004974X^2^ + 0.002006X + 1.0121, R^2^ = 0.7412, *p* < 0.0001, Y_max_ = X value of 17.7%) and PER (Y = 0.0000004873X^3^ − 0.000100X^2^ + 0.003680X + 1.7915, R^2^ = 0.8589, *p* < 0.0001, Y_max_ = X value of 21.9%) in regression analysis, respectively. PR of fish varied from 33.71 to 34.78% (34.04 ± 0.761%), but it was not statistically (*p* > 0.3) different among the experimental feeds replacing various levels of FM with CMC.

K of olive flounder changed from 1.04 to 1.11 g/cm^3^ (1.08 ± 0.045 g/cm^3^), VSI was 3.16% (3.16 ± 0.007%) for all diets, and HSI changed from 1.22 to 1.23% (1.22 ± 0.003%). None of the biological indices of fish were remarkably (*p* > 0.07, *p* > 0.8, and *p* > 0.8, respectively) affected by FM substitution with CMC in feeds.

### 3.4. Plasma Parameters of Fish

Plasma AST of olive flounder changed from 12.8 to 13.3 U/L (13.1 ± 1.94 U/L; mean ± SD), ALT was changed from 5.7 to 6.1 U/L (5.9 ± 1.17 U/L), ALP was changed from 91.2 to 92.4 U/L (91.8 ± 11.45 U/L), TBL was changed from 0.9 to 1.0 mg/dL (0.9 ± 0.022 mg/dL), TCO was changed from 179.4 to 183.4 mg/dL (180.9 ± 13.58 mg/dL), TRG was changed from 443.2 to 449.7 mg/dL (447.5 ± 25.92 mg/dL), TPT was changed from 3.5 to 3.7 g/dL (3.6 ± 0.031 g/dL), and ALB was changed from 1.0 to 1.1 g/dL (1.0 ± 0.13 g/dL) (Table 7). Plasma parameters of olive flounder were not statistically (*p* > 0.9, *p* > 0.9, *p* > 0.9, *p* > 0.9, *p* > 0.9, *p* > 0.9, *p* > 0.9, and *p* > 0.2, respectively) influenced by dietary treatments.

### 3.5. Serum Lysozyme Activity and SOD of Fish

Serum lysozyme activity of fish changed from 741.5 to 919.8 U/mL (833.9 ± 115.48%; mean ± SD), and SOD changed from 68.3 to 75.2% (71.6 ± 6.13%) (Table 8). Dietary treatments had no statistical (*p* > 0.4 and *p >* 0.5, respectively) impacts on these parameters. 

### 3.6. Biochemical Composition of the Whole-Body Fish

The moisture content of the whole-body fish changed from 74.9 to 75.4% (75.2 ± 0.28%; mean ± SD), crude protein content changed from 17.4 to 17.6% (17.5 ± 0.08%), crude lipid content changed from 3.1 to 3.4% (3.3 ± 0.11%), and ash content changed from 3.5 to 3.7% (3.6 ± 0.12%) (Table 9). These measurements were not statistically (*p* > 0.4, *p* > 0.1, *p* > 0.1, and *p* > 0.2, respectively) altered by dietary FM substitution by CMC.

The AA profiles of the whole-body olive flounder were not statistically (*p* > 0.05) changed by dietary treatments (Table 10).

The ∑SFA content of the whole-body fish ranged from 24.39 to 25.12% of total FA (24.81 ± 0.323% of total FA; mean ± SD), the ∑MUFA content of the whole-body fish ranged from 26.09 to 34.91% of total FA (30.20 ± 3.089% of total FA), and the ∑n−3HUFA content of the whole-body fish ranged from 9.29 to 15.33% of total FA (12.94 ± 2.236% of total FA). The ∑SFA content of the whole-body olive flounder fed the CMC80 and CMC100 diets was statistically (*p* < 0.01) higher than that of fish fed the CMC0 diet, but not statistically (*p* > 0.05) different from that of fish fed the CMC10, CMC20, CMC40, and CMC60 diets (Table 11). The ∑MUFA content in the whole-body fish linearly elevated with dietary increased FM substitution with CMC, but the ∑n−3 HUFA content in the whole-body olive flounder decreased.

### 3.7. Survival of Fish in the 8-Day Post-Observation after S. iniae Infection

**Figure 1:**
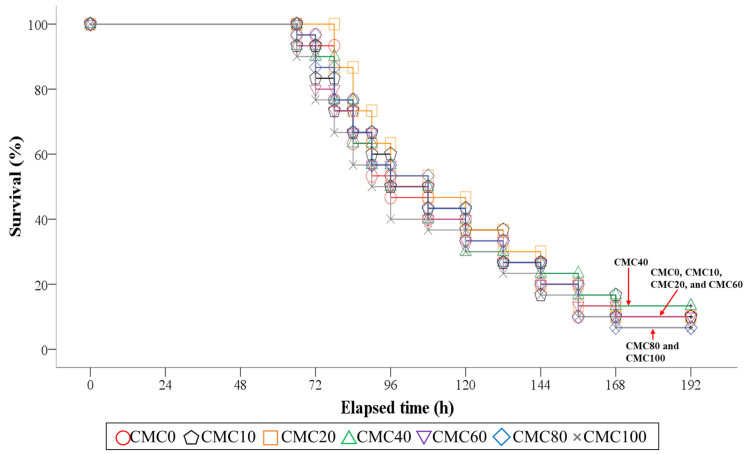
Kaplan–Meier survival curve (mean of triplicate ± SE) of juvenile olive flounder fed the experimental diets replacing various levels of fish meal with combined meat meal and chicken by-product meal (CMC) for 8 weeks, and then artificially infected with *S. iniae* in the following 8 days (*p* > 0.9 for Log-Rank and Wilcoxon test). CMC: combined meat meal and chicken by-product meal; CMC0: control diet containing 65% fish meal; CMC10: dietary 10% fish meal substitution with CMC; CMC20: dietary 20% fish meal substitution with CMC; CMC40: dietary 40% fish meal substitution with CMC; CMC60: dietary 60% fish meal substitution with CMC; CMC80: dietary 80% fish meal substitution with CMC; CMC100: dietary 100% fish meal substitution with CMC.

Figure 1 presents the survival of olive flounder during the 8-day post-observation after *S. iniae* infection after the 8-week feeding trial. Olive flounder fed all experimental diets except for the CMC20 diet started to exhibit mortality in 66 h after *S. iniae* infection. However, the experimental diets did not statistically (*p* > 0.9 for Log-Rank and Wilcoxon test) change the survival of olive flounder, and the mean survival of olive flounder was 9.5% at the end of the 8-day post-observation after *S. iniae* infection.

## 4. Discussion

All EAA contents in FM, except for arginine, were relatively high over those in CMC, resulting in increased arginine content, but decreased for all EAA in the experimental diets with increasing FM substitution levels with CMC. Arginine (2.04–2.10% of the diet) [45], lysine (1.50–2.10% of the diet) [46], and threonine (1.03% of the diet) [47] requirements of olive flounder were met in all experimental feeds in this study. However, the methionine requirement (1.44–1.49% of the diet in the presence of 0.06% cysteine) of olive flounder [48] seemed not to be met in all experimental feeds, including the CMC0 diet. However, previous studies have demonstrated that cysteine could spare 40–50% of the dietary methionine requirements of fish [50,51], and relatively low content of methionine (0.53–1.14% of the diet) in the presence of high cysteine (0.37–0.63% of the diet) in the experimental feeds, except for diets where replacing a high amount of FM with CMC did not bring about deteriorated growth in this experiment. Furthermore, similar or superior SGR values (1.92–3.24%/day) of juvenile olive flounder in this study compared to those reported in similar sizes of olive flounder [8,9] might indicate that the growth performance of olive flounder was well achieved in this experiment.

No statistically discernible differences in weight gain and SGR of fish fed the CMC0 and CMC60 diets in the current study implied that CMC could substitute FM up to 60% in diets without causing unfavorable impacts on the growth performance of fish. Nevertheless, the greatest growth performances were found in olive flounder fed the CMC20 diet. Ha et al. [23] proved that the high (80% crude protein) quality of MM could replace FM up to 40% in an olive flounder diet containing 65% FM without producing deteriorated growth performances, feed consumption, or feed utilization when juvenile fish were fed with a 65% FM-basal diet or one of the diets substituting 10, 20, 40, 60, 80, and 100% FM by MM without AA supplementation for 8 weeks. They also stressed that a diet substituting 20% FM with MM achieved superior weight gain and SGR to a 65% FM-basal diet or all other MM-substituted diets. Sato and Kikuchi [24] unveiled that FM up to 60% can be replaceable with MM without compromising the growth of olive flounder when fish were fed with an 80% FM-based diet or one of the diets replacing various (20, 40, 60, 80, and 100%) levels of FM with high-quality MM (ca. 80% crude protein) with limiting AA (lysine, methionine, and tryptophan) supplementation. However, low (ca. 65% crude protein) quality of MM can substitute FM up to 20% in 65% FM-based feed without causing unfavorable effects on growth and feed availability of olive flounder when fish fed with a 65% FM-based feed or one of feeds substituting graded (10, 20, 30, 40, and 50%) levels of FM by MM without AA supplementation for 8 weeks [14]. The substitutability of FM with MM in the olive flounder feeds seemed to be affected by the quality of MM and/or EAA supplementation. CBM could substitute 50% FM without causing deteriorated growth of olive flounder when juvenile fish were fed with a 65% FM-basal diet or diets substituting graded (10–50%) levels of FM by CBM [28]. Dietary FM up to 28.6% could be replaced with chicken waste meal without deteriorating the growth and FE of seabass when seabass were fed with a 35% FM-basal diet or one of the diets substituting 14.3, 28.6, 42.9, and 57.1% FM with chicken waste meal for 56 days [29]. However, animal proteins should be utilized after necessary treatment and stabilization since they might generate chemical or microbiological contaminants during the processing process [52,53].

The weight gain and SGR of olive flounder fed the CMC10, CMC20, and CMC40 diets were superior to those of fish fed the CMC0 diet. Furthermore, higher substitutability of FM up to 60% (39% FM protein in the diet) with CMC in this study compared to that of FM up to 40% (26% FM protein in the diet) and 50% (32.5% FM protein in the diet) with MM [23] and CBM [28], respectively, in the 65% FM-basal diets of olive flounder could result from the synergetic effect of the CMC as a substitute for FM. Therefore, the use of combined proteins as a FM substitute can effectively address improvements in nutritional EAA. All experimental conditions, except for the alternative sources for FM in the olive flounder feeds [CMC in this study vs. MM in Ha et al.’s [23] study], were identical. EAA content, except for arginine and tryptophan in CMC in this study, is higher than that in MM [23]. When compared to the same FM replacement levels with either CMC or MM in the olive flounder diets, dietary CMC replacement led to higher isoleucine, leucine, phenylalanine, threonine, and valine content than dietary MM replacement. Therefore, a higher (39% FM protein) amount of FM protein can be substituted with CMC in the feeds of olive flounder in this study than MM (26% FM protein in the diet) in Ha et al.’s [23] study because of compensated EAA in the former. Likewise, Gunathilaka et al. [32] demonstrated that the combined MM and CBM were effective substitutes for FM in red sea bream feeds, substituting 50% FM with soy protein concentrate, corn gluten meal, MM, CBM, and their combinations. Similarly, combined animal proteins can replace FM up to 80% in a 70% FM-basal diet without causing any negative impact on the weight gain of hybrid groupers when fish were fed with a 70% FM-basal diet or one of the diets replacing 20, 40, 60, and 80% FM with combined animal proteins (PBM, SBM, and shrimp meal) for 8 weeks [15].

Marine fish generally require higher n−3 HUFA, such as EPA and DHA, than C18 polyunsaturated FA, such as linoleic and linolenic acids, in their diets [54]. The ∑SFA and ∑MUFA in the experimental diets elevated with dietary increased FM replacement with CMC but decreased for the ∑n−3 HUFA. Lower ∑n−3 HUFA content (4.90 and 3.26% of total FA) in the CMC80 and CMC100 diets than the ∑n−3 HUFA requirement in the olive flounder diet [49] could demonstrate why olive flounder fed the CMC80 and CMC100 diets attained inferior growth compared to olive flounder fed all other diets in this study. Low ∑n−3 HUFA content in the CMC80 and CMC100 diets resulted from lower fish oil to formulate isolipidic diets and lower ∑n−3 HUFA content in CMC compared to FM. Fish oil is rich in n−3 HUFA, including EPA and DHA [55,56], while MM [16,23] and CBM [57] have lower EPA and DHA than FM.

The optimal replacement levels of CMC for FM in the experimental feeds in this experiment were estimated to be 24.1, 24.4, 24.0, 17.7, and 21.9% based on regression analysis of weight gain, SGR, feed consumption, FE, and PER, respectively. These results were consistent with the output of multiple comparisons of variables, exhibiting that the greatest growth performance and highest feed consumption were attained in olive flounder fed the CMC20 diet. However, the CMC60 diet seemed to be the most desirable feeding treatment for farmers in terms of lowering feed costs, since the price (USD 1.06/kg CMC, 1 USD = 1200 KRW) of CMC is about half the price (USD 2.17/kg FM) of FM.

Improvements in the growth performance of olive flounder were directly attributed to improvements in feed consumption in this experiment. CMC had higher arginine content than FM, and elevated FM substitution levels with CMC in the experimental diets increased arginine content. Arginine acts as a feed attractant and/or stimulant in some fish species [58,59]. However, several studies also demonstrated that exceeding arginine levels in diets deteriorated the growth performance and feed consumption of fish [60,61]. The highest FE was found in olive flounder fed the CMC20 diet. The FE and PER of olive flounder fed the CMC60 diet were similar to those of fish fed the CMC0 diet. This suggests that the CMC60 diet (39.0% FM protein in the diet) was an optimum substitution level for FM in the olive flounder diet without adverse effects on growth or feed availability. Likewise, several studies demonstrated that replacement of FM with various animal proteins did not negatively impact the growth and feed availability of olive flounder [35,57].

Several studies have demonstrated that low-FM diets frequently lead to reduced feed consumption in fish because of deteriorated feed palatability [62,63]. High (80 and 100%) FM substitution with CMC in diets led to deteriorated feed consumption by olive flounder, resulting in poor growth of olive flounder. Incorporating feed attractants and stimulants into developing low-FM feeds is a very effective way to improve the feed consumption of fish. For instance, jack mackerel meal exhibited the strongest feed attractant response to olive flounder [64] among various crude protein sources, and its incorporation in low-FM diets substituting various sources of animal and plant proteins for FM improved feed availability and growth of olive flounder [65,66]. Nevertheless, the effect of feed attractants and stimulants on the performance of fish may vary depending on the fish species [67].

The K is used as an indicator of health, obesity, and well-being in fish as it integrates several organizational processes and organosomatic indices, such as VSI and HSI, which are commonly used as stress-related indicators [29,68]. No difference in biological indices of olive flounder in this study might indicate that dietary FM replacements with CMC did not bring about a negative effect on K, VSI, and HSI. Likewise, K and HSI of the malabar grouper [33] and giant croaker (*Nibea japonica*) [69] were not influenced by dietary FM replacements with the blend of animal proteins. The biological indices of red sea bream were not changed by FM substitution with plants (soy concentrate meal and corn gluten meal), animals (MM and CBM), or their combinations in diets [32] as well.

Plasma parameters of fish are useful for examining the health status of fish [70]. Dietary FM replacements with CMC had no significant effects on plasma measurements (AST, ALT, ALP, TBL, TCO, TRG, TPT, and ALB) of olive flounder in this experiment. Likewise, dietary FM replacements with MM [14,23] and CBM [28] in the olive flounder diets did not influence plasma measurements, proving that replacement of FM with MM, CBM, and their combination in the diets did not adversely affect plasma parameters of olive flounder. In contrast, dietary increased FM substitution levels with the mixture of animal proteins changed plasma AST, ALT, and cholesterol levels because of increased HSI and crude lipid content of hybrid groupers when hybrid groupers were fed with a 70% FM-based diet or one of the diets substituting 20, 40, 60, and 80% FM with the mixture of animal proteins (PBM, shrimp meal, and blood meal) [15].

Non-specific immunological responses of fish are influenced by the nutritional condition of fish [71]. We assessed serum lysozyme activity and SOD of olive flounder because lysozyme activity is a significant effector for antibacterial immunity [72] and SOD regulates oxidative stress [73]. No differences in the serum lysozyme activity and SOD of olive flounder were found in this experiment, suggesting that complete (100%) FM replacement with CMC did not influence serum lysozyme activity and SOD. Likewise, SOD and lysozyme activity of olive flounder [14,23] and rockfish [16] were not affected by dietary FM substitution with MM. Unlike this study, however, dietary FM substitution with various animal and/or plant sources influenced the SOD and/or lysozyme activity of fish [32,57], and lower lysozyme activity and SOD were obtained in red sea bream and olive flounder, achieving inferior growth.

The biochemical composition of the whole-body olive flounder, except for the FA profiles, was not changed by dietary treatments in this study, indicating that complete FM substitution with CMC in diets did not alter the proximate composition or AA profiles of the whole-body olive flounder. Likewise, dietary FM replacement with CBM did not alter the chemical composition and AA profiles of olive flounder [28]. Wu et al. [69] demonstrated that the chemical composition of the whole-body giant croaker was not influenced by dietary FM replacement with the combined animal proteins. Unlike this study, however, complete FM substitution with PBM lowered the crude lipid content of the whole-body gilthead sea bream, which directly resulted from the lower (15.53%) content of crude lipid in the diet substituting 100% FM with PBM compared to a 58% FM-based diet (16.83% crude lipid content) [25]. Isonitrogenous (56.5%) and isolipidic (10.8%) feeds in this study did not affect the proximate composition of the whole-body olive flounder. Proximate composition and AA profiles, except for alanine, of the whole-body olive flounder were not changed by dietary FM replacement with MM [23]. No remarkable differences in AA profiles in the whole-body olive flounder can be demonstrated by Yamamoto et al.’s [74] study, in which specific AA profiles of fish can be similar regardless of diets because proteins are synthesized from the DNA information of the fish body. Increased FM replacement with CMC in diets led to increased ∑SFA and ∑MUFA, but decreased ∑n−3 HUFA in the whole-body fish in the current study, indicating that dietary FA profiles were well reflected in the whole-body FA profiles. Likewise, Dawson et al. [17] explained that dietary FM substitution with PBM impacted the FA profiles of the whole-body black sea bass (*Centropristis striata*). Similarly, FA profiles of whole-body olive flounder were changed when 30% FM was replaced with the various animal proteins in feeds [57]. 

FM replacement with an alternative protein source in fish diets might affect fish immunity [34,35], and the suitability of low-FM diets under commercial fish farm conditions, where fish are highly susceptible to various pathogens, is difficult to predict based on only a short-term feeding trial. Therefore, the disease resistance of fish against *S. iniae*, which contributes to the high mortality of olive flounder in aquaculture in Korea [37,38], after the 8-week feeding trial in this study might be helpful to assess the potential use of CMC as a FM replacer in the olive flounder diet. In the challenge test of this study, dead fish exhibited representative symptoms of *S. iniae* infection in olive flounder, such as the blackening of skin, hernia, and hemorrhagic sepsis [23,75]. However, there was no remarkable difference in the survival of fish at the end of the 8-day post-observation period after *S. iniae* infection, indicating that complete FM replacement with CMC in diets did not cause any negative impact on survival. Likewise, survival of olive flounder was not influenced by FM replacement with MM in diets after *S. iniae* infection when juvenile fish were fed with diets substituting various levels of FM with MM for 56 days and then infected with *S. iniae* [23]. Pham et al. [76] demonstrated that dietary 10% FM replacement with tuna viscera hydrolysate led to higher disease resistance of pompano (*Trachinotus blochii*) compared to a 63% FM-basal diet or dietary substitution of 20% FM with tuna visceral hydrolysate after *S. iniae* infection when juvenile fish were fed with a 63% FM-basal diet or one of the diets replacing 10 and 20% FM with tuna viscera hydrolysate for 10 weeks and then artificially infected with *S. iniae.* However, Sealey et al. [77] proved that 100% FM replacement with animal proteins in diets did not affect growth and disease resistance against *Flavobacterium psychrophilum* negatively when rainbow trout were fed with a 68.58% FM-based diet or one of the diets replacing 100% FM with various animal proteins (chicken concentrate, poultry by-product blend, or chicken and egg concentrate) for 56 days and then challenged by *F. psychrophilum*. No remarkable differences in plasma measurements and serum lysozyme activity and SOD at the completion of the 8-week feeding trial and disease resistance of olive flounder at the end of the 8-day post-observation period after *S. iniae* infection in this study indicated that diverse levels of FM replacement with CMC in diets did not mitigate plasma and serum parameters and disease resistance of olive flounder against *S. iniae*. 

These findings proved the potential of CMC as an effective FM replacer in the olive flounder diet, suggesting promising prospects for olive flounder aquaculture.

## 5. Conclusions

CMC can replace FM up to 60% (39% FM protein in the diet) in the 65% FM-based diet of olive flounder without deteriorating growth, feed availability, proximate composition, AA profiles, plasma and serum parameters, or disease resistance against *S. iniae*. The desirable effects of CMC as a replacer for FM were outstanding, especially in diets substituting 10, 20, and 40% FM with CMC on growth performance and feed consumption of olive flounder. Dietary optimum FM replacement levels with CMC to induce the maximum weight gain, SGR, feed consumption, FE, and PER of olive flounder were estimated to be 24.1, 24.4, 24.0, 17.7, and 21.9%, respectively, according to regression analysis.

## Figures and Tables

**Table 1:** Ingredients and proximate composition of the experimental diets (%, DM basis).

	Experimental Diets						
	CMC0	CMC10	CMC20	CMC40	CMC60	CMC80	CMC100
Ingredient (%, DM)							
Fish meal ^1^	65.0	58.5	52.0	39.0	26.0	13.0	0.0
Combined meat meal and chicken by-product meal (CMC) ^2^	0.0	6.1	12.2	25.0	36.7	49.0	61.2
Dehulled soybean meal	12.0	12.0	12.0	12.0	12.0	12.0	12.0
Wheat flour	14.9	15.7	16.4	18.3	19.3	20.7	22.3
Krill meal	1.0	1.0	1.0	1.0	1.0	1.0	1.0
Fish oil	2.3	2.3	2.3	2.3	2.3	1.8	1.0
Soybean oil	2.3	1.9	1.6	0.8	0.2	0.0	0.0
Vitamin premix ^3^	1.0	1.0	1.0	1.0	1.0	1.0	1.0
Mineral premix ^4^	1.0	1.0	1.0	1.0	1.0	1.0	1.0
Choline	0.5	0.5	0.5	0.5	0.5	0.5	0.5
Nutrients (%, DM)							
Dry matter	97.0 ± 0.27	96.8 ± 0.03	96.4 ± 0.21	95.8 ± 0.13	96.1 ± 0.23	94.1 ± 0.90	94.2 ± 0.04
Crude protein	56.0 ± 0.37	55.9 ± 0.78	56.6 ± 0.09	56.4 ± 0.35	56.6 ± 0.06	57.0 ± 0.40	57.0 ± 0.72
Crude lipid	10.8 ± 0.01	10.6 ± 0.34	10.8 ± 0.28	10.5 ± 0.08	10.6 ± 0.03	10.8 ± 0.31	10.8 ± 0.39
Ash	14.4 ± 0.00	13.9 ± 0.10	12.9 ± 0.19	11.0 ± 0.15	10.8 ± 0.11	9.0 ± 0.19	7.8 ± 0.39

Nutrient values are presented (mean of duplicate ± SD). ^1^ Fish meal (crude protein: 71.4%, crude lipid: 7.9%, and ash: 18.6%) was imported from Chile [USD 2.17/kg FM, 1 USD = 1200 KRW]. ^2^ Combined meat meal and chicken by-product meal (CMC) (crude protein: 73.7%, crude lipid: 13.9%, and ash: 8.7%) is the mixture of meat meal (MM; crude protein: 80.3%, crude lipid: 13.9%, and ash: 8.7%) and chicken by-product meal (CBM; crude protein: 67.1%, crude lipid: 13.9%, and ash: 8.7%) at the ratio of 1:1 and each ingredient was purchased from Daekyung Oil & Transportation Co., Ltd. (Busan, Republic of Korea) (USD 1.20/kg MM) and Charmfre Co., Ltd. (Buan-gun, Jeollabuk-do, Republic of Korea) (USD 0.92/kg CBM), respectively. ^3^ Vitamin premix (g/kg mix): L-ascorbic acid, 121.2; DL-α-tocopheryl acetate, 18.8; thiamin hydrochloride, 2.7; riboflavin, 9.1; pyridoxine hydrochloride, 1.8; niacin, 36.4; Ca-D-pantothenate, 12.7; myo-inositol, 181.8; D-biotin, 0.27; folic acid, 0.68; p-aminobenzoic acid, 18.2; menadione, 1.8; retinyl acetate, 0.73; cholecalciferol, 0.003; cyanocobalamin, 0.003. ^4^ Mineral premix (g/kg mix): MgSO_4_·7H_2_O, 80.0; NaH_2_PO_4_·2H_2_O, 370.0; KCl, 130.0; ferric citrate, 40.0; ZnSO_4_·7H_2_O, 20.0; Ca-lactate, 356.5; CuCl, 0.2; AlCl_3_·6H_2_O, 0.15; KI, 0.15; Na_2_Se_2_O_3_, 0.01; MnSO_4_·H_2_O, 2.0; CoCl_2_·6H_2_O, 1.0.

**Table 2:** Amino acid profiles (% of the diet) of the experimental diets.

	Ingredients				Requirement	Experimental Diets						
	FM	MM	CBM	CMC		CMC0	CMC10	CMC20	CMC40	CMC60	CMC80	CMC100
Essential amino acid (EAA) (%)												
Arginine	3.78	5.32	4.68	5.00	2.04–2.10 ^1^	3.03	3.14	3.43	3.74	3.82	3.92	3.99
Histidine	2.05	1.17	1.47	1.32		1.28	1.27	1.25	1.23	1.22	1.21	1.20
Isoleucine	2.83	1.66	2.81	2.24		1.95	1.93	1.92	1.89	1.88	1.86	1.85
Leucine	4.93	3.48	4.82	4.15		4.40	4.23	4.17	4.10	4.08	3.91	3.69
Lysine	5.14	3.56	4.43	4.00	1.50–2.10 ^2^	3.91	3.74	3.47	3.44	3.40	3.31	3.22
Methionine	1.84	0.86	1.45	1.16	1.44–1.49 ^3^	1.14	0.99	0.94	0.84	0.79	0.64	0.53
Phenylalanine	2.71	2.08	2.68	2.38		1.90	1.88	1.86	1.84	1.80	1.77	1.74
Threonine	2.87	2.00	2.76	2.38	1.03 ^4^	2.26	2.24	2.17	2.13	2.09	2.03	1.95
Tryptophan	0.71	0.53	0.50	0.52		0.54	0.48	0.47	0.46	0.43	0.40	0.33
Valine	3.44	2.61	3.33	2.97		2.14	2.10	2.07	2.03	2.00	1.98	1.93
∑EAA ^5^	30.30	23.27	28.93	26.10		22.55	22.00	21.75	21.70	21.51	21.03	20.43
Non-essential amino acid (NEAA) (%)												
Alanine	4.18	5.82	4.47	5.15		3.09	3.17	3.22	3.28	3.33	3.37	3.53
Aspartic acid	6.03	5.04	5.86	5.45		4.88	4.83	4.67	4.66	4.60	4.54	4.49
Cysteine	0.63	0.37	0.45	0.41		0.63	0.57	0.55	0.45	0.42	0.40	0.37
Glutamic acid	8.49	8.16	9.07	8.62		6.85	6.90	6.91	6.95	6.99	7.03	7.04
Glycine	4.19	12.26	5.82	9.04		3.08	3.44	3.91	4.49	5.17	5.71	6.45
Proline	2.59	7.34	3.82	5.58		3.83	3.95	4.02	4.18	4.46	5.25	6.04
Serine	2.57	2.59	2.52	2.56		1.96	1.95	1.93	1.92	1.90	1.87	1.86
Tyrosine	1.61	1.23	1.97	1.60		1.86	1.85	1.83	1.80	1.78	1.77	1.74
∑NEAA ^6^	30.29	42.81	33.98	38.40		26.18	26.66	27.04	27.73	28.65	29.94	31.52

CMC: combined meat meal and chicken by-product meal; CMC0: control diet containing 65% fish meal; CMC10: dietary 10% fish meal substitution with CMC; CMC20: dietary 20% fish meal substitution with CMC; CMC40: dietary 40% fish meal substitution with CMC; CMC60: dietary 60% fish meal substitution with CMC; CMC80: dietary 80% fish meal substitution with CMC; CMC100: dietary 100% fish meal substitution with CMC. ^1,2,3,4^ Data were obtained from Alam et al. [45], Forster and Ogata [46], Alam et al. [48], and Hasanthi et al.’s [47] studies, respectively. ^5^ ∑EAA: Total content of essential amino acids. ^6^ ∑NEAA: Total content of non-essential amino acids.

**Table 3:** Fatty acid profiles (%, total fatty acids) of the main protein sources and experimental diets.

	Ingredients				Requirement	Experimental Diets						
	FM	MM	CBM	CMC		CMC0	CMC10	CMC20	CMC40	CMC60	CMC80	CMC100
C14:0	4.16	2.60	1.41	2.01		3.00	2.98	2.82	2.56	2.19	2.13	2.05
C16:0	27.11	29.89	35.89	32.89		15.87	17.39	17.84	18.97	19.28	20.23	23.15
C18:0	9.44	12.27	13.57	12.92		5.70	6.43	6.46	7.14	8.66	10.13	13.29
∑SFA ^1^	40.71	44.76	50.87	47.82		24.57	26.80	27.12	28.67	30.13	32.49	38.49
C16:1n−7	9.26	7.88	5.22	6.55		3.41	3.38	3.30	3.11	2.96	2.56	2.42
C17:1n−7	0.59	0.47	0.29	0.38		0.52	0.50	0.49	0.45	0.42	0.38	0.34
C18:1n−9	17.33	34.37	37.59	35.98		19.86	22.51	24.73	29.60	31.23	35.27	38.49
C20:1n−9	1.12	0.62	0.22	0.42		1.32	0.96	0.92	0.90	0.85	0.81	0.57
∑MUFA ^2^	28.30	43.34	43.32	43.33		25.11	27.35	29.44	34.06	35.46	39.02	41.82
C18:2n−6	2.07	1.66		1.66		27.21	25.69	24.54	22.50	21.87	18.39	13.05
C18:3n−3	0.73	0.59	0.19	0.39		3.97	3.59	3.35	2.89	2.72	1.97	1.31
C20:4n−6	1.53	0.73	0.59	0.66		1.07	0.95	0.86	0.79	0.59	0.35	0.21
C20:5n−3	4.89					5.02	4.41	4.12	3.46	2.89	2.03	1.87
C22:5n−3	0.90	0.33	0.29	0.31		0.98	0.84	0.73	0.64	0.49	0.37	0.30
C22:6n−3	13.11					8.44	6.94	6.71	5.41	4.61	2.50	1.09
∑n−3 HUFA ^3^	18.90	0.33	0.29	0.31	7.41–9.26 ^4^	14.44	12.19	11.56	9.51	7.99	4.90	3.26
Unknown	7.76	8.59	4.74	5.84		3.63	3.43	3.13	1.58	1.24	2.88	1.86

CMC: combined meat meal and chicken by-product meal; CMC0: control diet containing 65% fish meal; CMC10: dietary 10% fish meal substitution with CMC; CMC20: dietary 20% fish meal substitution with CMC; CMC40: dietary 40% fish meal substitution with CMC; CMC60: dietary 60% fish meal substitution with CMC; CMC80: dietary 80% fish meal substitution with CMC; CMC100: dietary 100% fish meal substitution with CMC. ^1^ ∑SFA: Total content of saturated fatty acids. ^2^ ∑MUFA: Total content of monounsaturated fatty acids. ^3^ ∑n−3 HUFA: Total content of n−3 highly unsaturated fatty acids. ^4^ Data were obtained from Kim and Lee’s [49] study.

**Table 4:** Survival (%), weight gain (g/fish), and specific growth rate (SGR) of olive flounder fed the experimental diets for 8 weeks.

Experimental Diets	Initial Weight (g/fish)	Final Weight (g/fish)	Survival (%)	Weight Gain (g/fish)	SGR (%/day) ^1^
CMC0	9.2 ± 0.00	44.3 ± 0.96 ^c^	100 ± 0.00	35.1 ± 0.96 ^c^	2.81 ± 0.039 ^c^
CMC10	9.2 ± 0.00	49.2 ± 0.60 ^b^	97.3 ± 2.31	40.0 ± 0.60 ^b^	2.99 ± 0.021 ^b^
CMC20	9.2 ± 0.00	56.5 ± 2.58 ^a^	100 ± 0.00	47.3 ± 2.58 ^a^	3.24 ± 0.082 ^a^
CMC40	9.2 ± 0.01	49.4 ± 1.18 ^b^	100 ± 0.00	40.2 ± 1.17 ^b^	3.00 ± 0.040 ^b^
CMC60	9.2 ± 0.01	40.9 ± 1.63 ^c^	98.7 ± 2.31	31.7 ± 1.62 ^c^	2.66 ± 0.068 ^c^
CMC80	9.2 ± 0.01	33.9 ± 0.63 ^d^	97.3 ± 2.31	24.7 ± 0.63 ^d^	2.33 ± 0.033 ^d^
CMC100	9.2 ± 0.01	27.1 ± 1.31 ^e^	97.3 ± 2.31	17.8 ± 0.32 ^e^	1.92 ± 0.021 ^e^
*p*–value		*p* < 0.0001	*p* > 0.1	*p* < 0.0001	*p* < 0.0001

CMC: combined meat meal and chicken by-product meal; CMC0: control diet containing 65% fish meal; CMC10: dietary 10% fish meal substitution with CMC; CMC20: dietary 20% fish meal substitution with CMC; CMC40: dietary 40% fish meal substitution with CMC; CMC60: dietary 60% fish meal substitution with CMC; CMC80: dietary 80% fish meal substitution with CMC; CMC100: dietary 100% fish meal substitution with CMC. Values (mean of triplicate ± SD) in the same column sharing the same superscript letter are not significantly different (*p* > 0.05). ^1^ Specific growth rate (SGR, %/day) = [Ln final weight of fish (g) − Ln initial weight of fish (g)] × 100/days of the feeding trial.

**Table 5:** Effect of dietary fish meal replacement with combined meat meal and chicken by-product meal (independent variable) on weight gain, specific growth rate (SGR), feed consumption, feed efficiency (FE), and protein efficiency ratio (PER) of olive flounder (dependent variables) in the 8-week feeding trial.

Dependent Variables	Regression Analysis			
	Equation	*p*-Value	R^2^	Y_max_ (%)
Weight gain	Y = 0.000117X^3^ − 0.021899X^2^ + 0.852379X + 35.0989	0.0001	0.9560	24.1
SGR	Y = 0.000003662X^3^ − 0.000756X^2^ + 0.030304X + 2.8084	0.0001	0.9711	24.4
Feed consumption	Y = 0.000102X^3^ − 0.019279X^2^ + 0.748248X + 35.0246	0.0001	0.9608	24.0
FE	Y = −0.0000002692X^3^ − 0.00004974X^2^ + 0.002006X + 1.0121	0.0001	0.7412	17.7
PER	Y = 0.0000004873X^3^ − 0.000100X^2^ + 0.003680X + 1.7915	0.0001	0.8589	21.9

**Table 6:** Feed consumption, feed efficiency (FE), protein efficiency ratio (PER), protein retention (PR), condition factor (K), viscerosomatic index (VSI), and hepatosomatic index (HSI) of olive flounder fed the experimental diets for 8 weeks.

Experimental Diets	Feed Consumption (g/fish)	FE ^1^	PER ^2^	PR ^3^ (%)	K ^4^ (g/cm^3^)	VSI ^5^ (%)	HIS ^6^ (%)
CMC0	33.75 ± 0.544 ^c^	1.04 ± 0.012 ^ab^	1.85 ± 0.021 ^a^	34.78 ± 0.632	1.08 ± 0.018	3.16 ± 0.004	1.23 ± 0.000
CMC10	39.57 ± 0.542 ^b^	1.04 ± 0.022 ^ab^	1.81 ± 0.011 ^a^	33.73 ± 0.127	1.10 ± 0.003	3.16 ± 0.015	1.22 ± 0.000
CMC20	45.16 ± 0.760 ^a^	1.05 ± 0.040 ^a^	1.85 ± 0.071 ^a^	34.31 ± 1.327	1.11 ± 0.007	3.16 ± 0.011	1.22 ± 0.003
CMC40	39.43 ± 0.930 ^b^	1.02 ± 0.012 ^ab^	1.81 ± 0.022 ^a^	33.90 ± 0.400	1.11 ± 0.019	3.16 ± 0.001	1.23 ± 0.000
CMC60	31.60 ± 1.080 ^cd^	1.02 ± 0.007 ^ab^	1.79 ± 0.034 ^ab^	34.03 ± 0.389	1.06 ± 0.055	3.16 ± 0.002	1.22 ± 0.001
CMC80	25.27 ± 0.689 ^d^	1.00 ± 0.006 ^ab^	1.74 ± 0.029 ^ab^	33.71 ± 0.468	1.04 ± 0.057	3.16 ± 0.005	1.22 ± 0.005
CMC100	18.99 ± 0.592 ^e^	0.99 ± 0.020 ^b^	1.69 ± 0.074 ^b^	33.79 ± 1.303	1.04 ± 0.063	3.16 ± 0.008	1.22 ± 0.006
*p*–value	*p* < 0.0001	*p* < 0.03	*p* < 0.002	*p* > 0.3	*p* > 0.07	*p* > 0.8	*p* > 0.8

CMC: combined meat meal and chicken by-product meal; CMC0: control diet containing 65% fish meal; CMC10: dietary 10% fish meal substitution with CMC; CMC20: dietary 20% fish meal substitution with CMC; CMC40: dietary 40% fish meal substitution with CMC; CMC60: dietary 60% fish meal substitution with CMC; CMC80: dietary 80% fish meal substitution with CMC; CMC100: dietary 100% fish meal substitution with CMC. Values (mean of triplicate ± SD) in the same column sharing the same superscript letter are not significantly different (*p* > 0.05). ^1^ Feed efficiency (FE) = weight gain of fish/feed consumption. ^2^ Protein efficiency ratio (PER) = weight gain of fish/protein consumption. ^3^ Protein retention (PR, %) = protein gain of fish × 100/protein consumption. ^4^ Condition factor (K, g/cm^3^) = body weight of fish (g) × 100/total length of fish (cm^3^). ^5^ Viscerosomatic index (VSI, %) = viscera weight of fish × 100/body weight of fish. ^6^ Hepatosomatic index (HSI, %) = liver weight of fish × 100/body weight of fish.

**Table 7:** Plasma parameters of olive flounder fed the experimental diets for 8 weeks.

Experimental Diets	AST (U/L)	ALT (U/L)	ALP (U/L)	TBL (mg/dL)	TCO (mg/dL)	TRG (mg/dL)	TPT (g/dL)	ALB (g/dL)
CMC0	13.0 ± 1.22	5.7 ± 0.71	92.3 ± 4.66	0.9 ± 0.15	179.4 ± 16.71	443.2 ± 14.86	3.7 ± 0.35	1.1 ± 0.15
CMC10	13.0 ± 2.00	5.9 ± 1.05	92.4 ± 10.63	0.9 ± 0.28	180.4 ± 10.56	448.2 ± 26.48	3.5 ± 0.43	1.0 ± 0.11
CMC20	13.2 ± 2.49	6.1 ± 1.27	91.2 ± 9.04	0.9 ± 0.22	180.0 ± 20.67	449.4 ± 29.48	3.6 ± 0.28	1.0 ± 0.14
CMC40	13.3 ± 2.12	6.0 ± 0.87	92.1 ± 12.40	0.9 ± 0.29	180.4 ± 11.42	449.2 ± 28.27	3.6 ± 0.22	1.1 ± 0.12
CMC60	13.0 ± 2.29	5.9 ± 1.54	91.6 ± 12.85	1.0 ± 0.17	182.2 ± 9.65	449.7 ± 35.31	3.5 ± 0.20	1.0 ± 0.09
CMC80	13.2 ± 1.92	6.0 ± 1.32	91.7 ± 16.29	0.9 ± 0.22	180.4 ± 15.44	445.2 ± 24.09	3.5 ± 0.35	1.0 ± 0.12
CMC100	12.8 ± 1.92	5.9 ± 1.54	91.3 ± 14.61	0.9 ± 0.21	183.4 ± 11.64	447.7 ± 27.40	3.6 ± 0.34	1.0 ± 0.15
*p*–value	*p* > 0.9	*p* > 0.9	*p* > 0.9	*p* > 0.9	*p* > 0.9	*p* > 0.9	*p* > 0.9	*p* > 0.2

CMC: combined meat meal and chicken by-product meal; CMC0: control diet containing 65% fish meal; CMC10: dietary 10% fish meal substitution with CMC; CMC20: dietary 20% fish meal substitution with CMC; CMC40: dietary 40% fish meal substitution with CMC; CMC60: dietary 60% fish meal substitution with CMC; CMC80: dietary 80% fish meal substitution with CMC; CMC100: dietary 100% fish meal substitution with CMC. AST: aspartate aminotransferase; ALT: alanine aminotransferase; ALP: alkaline phosphatase; TBL: total bilirubin; TCO: total cholesterol; TRG: triglyceride; TPT: total protein; ALB: albumin.

**Table 8:** Serum lysozyme activity and superoxide dismutase (SOD) of olive flounder fed the experimental diets for 8 weeks.

Experimental Diets	Lysozyme Activity (U/mL)	SOD (%)
CMC0	741.5 ± 79.91	69.3 ± 5.01
CMC10	878.9 ± 93.41	70.8 ± 5.39
CMC20	857.0 ± 206.31	75.2 ± 6.52
CMC40	754.2 ± 36.66	70.4 ± 4.67
CMC60	919.8 ± 40.51	73.9 ± 7.62
CMC80	801.0 ± 164.46	73.6 ± 10.27
CMC100	884.9 ± 53.8	68.3 ± 5.25
*p*–value	*p* > 0.4	*p* > 0.5

CMC: combined meat meal and chicken by-product meal; CMC0: control diet containing 65% fish meal; CMC10: dietary 10% fish meal substitution with CMC; CMC20: dietary 20% fish meal substitution with CMC; CMC40: dietary 40% fish meal substitution with CMC; CMC60: dietary 60% fish meal substitution with CMC; CMC80: dietary 80% fish meal substitution with CMC; CMC100: dietary 100% fish meal substitution with CMC.

**Table 9:** Proximate composition (%, wet weight) of the whole-body olive flounder fed the experimental diets for 8 weeks.

Experimental Diets	Moisture	Crude Protein	Crude Lipid	Ash
CMC0	75.2 ± 0.26	17.4 ± 0.14	3.3 ± 0.01	3.7 ± 0.04
CMC10	74.9 ± 0.56	17.5 ± 0.04	3.2 ± 0.09	3.6 ± 0.13
CMC20	75.3 ± 0.18	17.5 ± 0.06	3.4 ± 0.09	3.7 ± 0.18
CMC40	75.2 ± 0.09	17.6 ± 0.04	3.1 ± 0.14	3.5 ± 0.09
CMC60	75.4 ± 0.24	17.5 ± 0.07	3.2 ± 0.07	3.6 ± 0.11
CMC80	75.1 ± 0.14	17.4 ± 0.03	3.3 ± 0.11	3.7 ± 0.07
CMC100	75.2 ± 0.19	17.5 ± 0.07	3.4 ± 0.14	3.5 ± 0.12
*p*–value	*p* > 0.4	*p* > 0.1	*p* > 0.1	*p* > 0.2

CMC: combined meat meal and chicken by-product meal; CMC0: control diet containing 65% fish meal; CMC10: dietary 10% fish meal substitution with CMC; CMC20: dietary 20% fish meal substitution with CMC; CMC40: dietary 40% fish meal substitution with CMC; CMC60: dietary 60% fish meal substitution with CMC; CMC80: dietary 80% fish meal substitution with CMC; CMC100: dietary 100% fish meal substitution with CMC.

**Table 10:** Amino acid profiles (%, wet weight) of the whole-body olive flounder fed the experimental diets for 8 weeks.

	Experimental Diets							
	CMC0	CMC10	CMC20	CMC40	CMC60	CMC80	CMC100	*p*–Value
Essential amino acid (EAA) (%)								
Arginine	0.35 ± 0.044	0.36 ± 0.040	0.37 ± 0.045	0.37 ± 0.025	0.39 ± 0.040	0.40 ± 0.030	0.41 ± 0.040	*p* > 0.4
Histidine	0.36 ± 0.038	0.35 ± 0.031	0.33 ± 0.032	0.33 ± 0.029	0.31 ± 0.040	0.30 ± 0.030	0.29 ± 0.045	*p* > 0.2
Isoleucine	0.69 ± 0.038	0.69 ± 0.040	0.68 ± 0.045	0.67 ± 0.040	0.66 ± 0.035	0.66 ± 0.055	0.65 ± 0.045	*p* > 0.8
Leucine	1.16 ± 0.006	1.15 ± 0.020	1.15 ± 0.025	1.14 ± 0.035	1.13 ± 0.030	1.13 ± 0.025	1.12 ± 0.045	*p* > 0.5
Lysine	0.51 ± 0.068	0.49 ± 0.025	0.49 ± 0.015	0.48 ± 0.045	0.47 ± 0.045	0.47 ± 0.035	0.46 ± 0.035	*p* > 0.9
Methionine	0.46 ± 0.021	0.44 ± 0.020	0.42 ± 0.030	0.41 ± 0.025	0.41 ± 0.040	0.40 ± 0.020	0.39 ± 0.025	*p* > 0.1
Phenylalanine	0.63 ± 0.012	0.61 ± 0.025	0.60 ± 0.050	0.59 ± 0.025	0.58 ± 0.040	0.56 ± 0.045	0.55 ± 0.035	*p* > 0.2
Threonine	0.73 ± 0.030	0.73 ± 0.030	0.71 ± 0.025	0.70 ± 0.030	0.70 ± 0.035	0.69 ± 0.035	0.69 ± 0.030	*p* > 0.2
Tryptophan	0.15 ± 0.029	0.15 ± 0.025	0.14 ± 0.040	0.14 ± 0.020	0.13 ± 0.025	0.13 ± 0.030	0.12 ± 0.025	*p* > 0.7
Valine	0.78 ± 0.038	0.76 ± 0.040	0.75 ± 0.025	0.74 ± 0.040	0.74 ± 0.025	0.73 ± 0.025	0.71 ± 0.040	*p* > 0.2
Non-essential amino acid (NEAA) (%)								
Alanine	1.17 ± 0.044	1.19 ± 0.035	1.20 ± 0.035	1.21 ± 0.025	1.23 ± 0.040	1.23 ± 0.035	1.25 ± 0.035	*p* > 0.1
Aspartic acid	1.11 ± 0.047	1.09 ± 0.035	1.07 ± 0.040	1.07 ± 0.050	1.06 ± 0.040	1.04 ± 0.035	1.03 ± 0.035	*p* > 0.2
Cysteine	0.19 ± 0.010	0.17 ± 0.030	0.16 ± 0.030	0.14 ± 0.045	0.14 ± 0.030	0.13 ± 0.030	0.12 ± 0.030	*p* > 0.1
Glutamic acid	2.19 ± 0.061	2.21 ± 0.035	2.23 ± 0.025	2.24 ± 0.030	2.24 ± 0.035	2.26 ± 0.035	2.27 ± 0.025	*p* > 0.2
Glycine	1.37 ± 0.136	1.39 ± 0.040	1.41 ± 0.055	1.41 ± 0.006	1.41 ± 0.062	1.42 ± 0.035	1.44 ± 0.055	*p* > 0.9
Proline	0.82 ± 0.026	0.82 ± 0.031	0.83 ± 0.025	0.85 ± 0.015	0.85 ± 0.020	0.86 ± 0.030	0.87 ± 0.030	*p* > 0.1
Serine	0.67 ± 0.032	0.67 ± 0.035	0.65 ± 0.025	0.64 ± 0.031	0.64 ± 0.035	0.63 ± 0.035	0.63 ± 0.035	*p* > 0.5
Tyrosine	0.39 ± 0.058	0.38 ± 0.045	0.38 ± 0.050	0.36 ± 0.035	0.35 ± 0.025	0.35 ± 0.020	0.34 ± 0.045	*p* > 0.6

CMC: combined meat meal and chicken by-product meal; CMC0: control diet containing 65% fish meal; CMC10: dietary 10% fish meal substitution with CMC; CMC20: dietary 20% fish meal substitution with CMC; CMC40: dietary 40% fish meal substitution with CMC; CMC60: dietary 60% fish meal substitution with CMC; CMC80: dietary 80% fish meal substitution with CMC; CMC100: dietary 100% fish meal substitution with CMC.

**Table 11:** Fatty acid profiles (%, total fatty acids) of the whole-body olive flounder fed the experimental diets for 8 weeks.

	Experimental Diets							
	CMC0	CMC10	CMC20	CMC40	CMC60	CMC80	CMC100	*p*–Value
C14:0	3.25 ± 0.050 ^a^	3.22 ± 0.047 ^ab^	3.17 ± 0.079 ^abc^	3.16 ± 0.040 ^abc^	3.14 ± 0.030 ^abc^	3.08 ± 0.075 ^bc^	3.04 ± 0.036 ^c^	*p* < 0.004
C16:0	16.56 ± 0.400 ^c^	16.73 ± 0.097 ^bc^	16.80 ± 0.056 ^abc^	16.96 ± 0.061 ^abc^	17.11 ± 0.176 ^ab^	17.23± 0.095 ^a^	17.28 ± 0.032 ^a^	*p* < 0.001
C18:0	4.58 ± 0.065 ^b^	4.64 ± 0.060 ^ab^	4.69 ± 0.051 ^ab^	4.71 ± 0.114 ^ab^	4.74 ± 0.062 ^ab^	4.78 ± 0.049 ^a^	4.80 ± 0.072 ^a^	*p* < 0.03
∑SFA ^1^	24.39 ± 0.515 ^b^	24.59 ± 0.180 ^ab^	24.66 ± 0.148 ^ab^	24.83 ± 0.083 ^ab^	24.99 ± 0.150 ^ab^	25.09 ± 0.081 ^a^	25.12 ± 0.075 ^a^	*p* < 0.01
C14:1n−5	0.19 ± 0.010	0.17 ± 0.030	0.16 ± 0.031	0.16 ± 0.015	0.14 ± 0.006	0.15 ± 0.025	0.14 ± 0.029	*p* > 0.1
C16:1n−7	4.00 ± 0.015 ^a^	3.99 ± 0.070 ^ab^	3.97 ± 0.031 ^ab^	3.94 ± 0.042 ^ab^	3.94 ± 0.056 ^ab^	3.92 ± 0.050 ^ab^	3.88 ± 0.021 ^b^	*p* < 0.03
C17:1n−7	0.60 ± 0.045	0.58 ± 0.050	0.57 ± 0.040	0.57 ± 0.021	0.55 ± 0.040	0.54 ± 0.031	0.53 ± 0.020	*p* > 0.3
C18:1n−9	20.95 ± 0.095 ^g^	22.54 ± 0.154 ^f^	23.07 ± 0.176 ^e^	24.62 ±0.076 ^d^	26.46 ± 0.090 ^c^	28.70 ± 0.122 ^b^	30.05 ± 0.108 ^a^	*p* < 0.0001
C20:1n−9	0.35 ± 0.010	0.35 ± 0.015	0.34 ± 0.015	0.33 ± 0.030	0.33 ± 0.006	0.32 ± 0.026	0.31 ± 0.020	*p* > 0.1
∑MUFA ^2^	26.09 ± 0.065 ^g^	27.64 ± 0.249 ^f^	28.12 ± 0.115 ^e^	29.61 ± 0.091 ^d^	31.42 ± 0.136 ^c^	33.63 ± 0.140 ^b^	34.91 ± 0.122 ^a^	*p* < 0.0001
C18:2n−6	21.24 ± 0.170 ^a^	21.08 ± 0.072 ^ab^	21.01 ± 0.100 ^ab^	20.87 ± 0.080 ^bc^	20.71 ± 0.074 ^cd^	20.63 ± 0.125 ^cd^	20.52 ± 0.080 ^d^	*p* < 0.0001
C18:3n−3	4.48 ± 0.100 ^a^	4.46 ± 0.107 ^a^	4.25 ± 0.064 ^ab^	4.16 ± 0.121 ^bc^	3.91 ± 0.114 ^cd^	3.74 ± 0.036 ^de^	3.62 ± 0.066 ^e^	*p* < 0.0001
C20:2n−6	1.56 ± 0.035	1.55 ± 0.050	1.54 ± 0.045	1.52 ± 0.060	1.51 ± 0.076	1.50 ± 0.035	1.48± 0.026	*p* > 0.4
C20:3n−3	0.26 ± 0.006	0.24 ± 0.006	0.24 ± 0.038	0.23 ± 0.035	0.23 ± 0.030	0.22 ± 0.025	0.22 ± 0.035	*p* > 0.9
C20:3n−6	0.74 ± 0.015	0.74 ± 0.031	0.73 ± 0.046	0.73 ± 0.035	0.72 ± 0.032	0.72 ± 0.046	0.71 ± 0.047	*p* > 0.6
C20:4n−6	1.11 ± 0.015 ^a^	1.08 ± 0.085 ^ab^	1.05 ± 0.050 ^ab^	1.00 ± 0.055 ^ab^	0.97 ± 0.035 ^abc^	0.94 ± 0.060 ^bc^	0.84 ± 0.050 ^c^	*p* < 0.0001
C20:5n−3	4.96 ± 0.035 ^a^	4.71 ± 0.144 ^ab^	4.65 ± 0.035 ^b^	4.58 ± 0.125 ^bc^	4.33 ± 0.119 ^c^	3.53 ± 0.035 ^d^	3.39 ± 0.131 ^d^	*p* < 0.0001
C22:5n−3	1.54 ± 0.015 ^a^	1.48 ± 0.079 ^ab^	1.43 ± 0.031 ^abc^	1.39 ± 0.029 ^abcd^	1.33 ± 0.076 ^bcd^	1.29 ± 0.057 ^cd^	1.26 ± 0.089 ^d^	*p* < 0.0001
C22:6n−3	8.56 ± 0.175 ^a^	8.39 ± 0.171 ^ab^	7.99 ± 0.125 ^b^	7.53 ± 0.100 ^c^	6.58 ± 0.159 ^d^	5.28 ± 0.091 ^e^	4.41 ± 0.163 ^f^	*p* < 0.0001
∑n−3 HUFA ^3^	15.33 ± 0.230 ^a^	14.82 ± 0.229 ^b^	14.31 ± 0.110 ^c^	13.73 ± 0.190 ^d^	12.47 ± 0.104 ^e^	10.32 ± 0.046 ^f^	9.29 ± 0.100 ^g^	*p* < 0.0001
Unknown	5.06 ± 0.115	4.05 ± 0.192	4.01 ± 0.168	3.54 ± 0.297	3.30 ± 0.267	3.43 ± 0.053	3.52 ± 0.045	

CMC: combined meat meal and chicken by-product meal; CMC0: control diet containing 65% fish meal; CMC10: dietary 10% fish meal substitution with CMC; CMC20: dietary 20% fish meal substitution with CMC; CMC40: dietary 40% fish meal substitution with CMC; CMC60: dietary 60% fish meal substitution with CMC; CMC80: dietary 80% fish meal substitution with CMC; CMC100: dietary 100% fish meal substitution with CMC. Values (mean of triplicate ± SD) in the same row sharing the same superscript letter are not significantly different (*p* > 0.05). ^1^ ∑SFA: Total content of saturated fatty acids. ^2^ ∑MUFA: Total content of monounsaturated fatty acids. ^3^ ∑n−3 HUFA: Total content of n−3 highly unsaturated fatty acids.

## Data Availability

Data are available on request from the authors.
